# Extracts from a Paper on “Plaster Splints in the Treatment of Fractures”

**Published:** 1885-09

**Authors:** 

**Affiliations:** Edgar, Ont.


### Extracts from a Paper on "Plaster Splints in the Treatment of Fractures," read before the Ontario Medical Association by Dr. Powell, of Edgar, Ont.

Remarking that only the finest and freshest dental plaster should be used in applying a plaster splint or bandage, the writer proceeds to direct the method of application of the spiral bandage of crinoline or gauze cloth, with plaster in the meshes, and to consider the uses of the "posterior plaster splint," and the risks and advantages of these methods: "The limb, if hairy, is to be oiled, and then it is to be enveloped in a thick layer of cotton batting taken unbroken from a roll. This layer should extend from the toes to the mid-thigh, should meet in the middle line in front and should be held in position by thread wound around the leg. Over this is to be applied, with moderate firmness, by the figure of 8 turn, and without reverses, a dry cotton roller. This must not be confounded with that relict of the dark ages, a "primary bandage." The gauze cloth of crinoline bandages before described are to be next placed, two at a time, on end in a bowl of warm water. When the bubbles cease to escape, they are taken out pressed to expel surplus water, and are applied from the toes up. No one turn should be drawn more snugly than another. If too tight, there is danger of strangulation, while, if too loose, the risk is that co-aptation of the fragments will not be maintained. Each layer as applied should be well rubbed by the hand to expel the air between it and the one next beneath it. From three to six layers will be required. An assistant is to make moderate extension during the application, and for from ten to thirty minutes afterwards. The heel should rest in his right palm, while his left hand is passed around the instep. If seated comfortably, and able to rest his forearm against his knees, he will be able to hold the foot at right angles with the limb, prevent rotary displacement by sighting over the great toe and inner margin of the patella, and will not become unsteady through muscular fatigue. To bring the toes up after the dressing is completed, is dangerous, since it is apt to constrict at the instep. To allow them to drop, is to run the risk of having the heel permanently elevated. When the limb is a heavy one, an inch wide stiffener of perforated tin may with advantage be interwoven in the bandage at each side. When the shell has become fairly firm, the limb is to be placed upon a moulded pillow, on which, in from two to four hours, it will become sufficiently rigid to stand being suspended. Such an apparel, light, shapely and perfectly fulfilling the indications, is the one I ordinarily employ in the *later* stages of leg fractures. It gives a firmer support than any hinged splint, and its use will materially shorten the period of confinement to bed. An elevated shoe on the sound side will assist the patient to keep the promise exacted from him that he will not, till allowed, bear weight on the injured limb while moving about on crutches.

In recent fracture, I much prefer an apparel that will allow examination from day to day till the consolidation is well advanced. Even after the most perfect reduction, I want to *know* and not simply to *hope* that the fragments maintain their proper position, and that the soft parts over them are in good condition. In 1872, I first applied what is known as the Bavarian or book splint. After using it a few times, I began to substitute shaped pieces of cheese-cloth saturated in plaster cream and placed between the inner and outer flannels, for the thick usual layer of solid plaster. With this hinged splint, I have treated about thirty fractures of the bones of the leg. It is lighter and stronger than the Bavarian, as ordinarily made, and it can be applied with safety when to use a plaster bandage would be malpractice. By placing the limb first on one side and then on the other, the halves of the splint can be raised like lids, and the seat of injury examined without risking in the least a disturbance of the process of repair. I show you one of these, but shall not urge its claims upon you since I wish to use the time at my disposal in advocating a still better and more easily applied retentive apparatus. This is one that I first saw at the Boston City Hospital three years ago. It is known as the "plaster posterior splint," and its development is largely due to the skill and ingenuity of Dr. R. A. Kingman, of Boston. This gentleman writes me that the original idea came from Brooklyn, N.Y., and that his connection with it has been in improving its details and demonstrating its utility and practicability. Through his courtesy, I am able to show you three photographs of one of these as applied before the Massachusetts Medical Society last year, after the reading of a paper on the subject by Dr. Geo. W.

Gay, surgeon to the City Hospital. I show you also a completed splint and a pattern of the shape into which the material for it was cut. One surgeon, well able to judge, considers this to be the most important advance in the treatment of leg fractures within the last fifty years. Another, and with him I certainly agree, thinks that it comes nearer than any other to being an ideal dressing for a broken leg. Unlike the Bavarian, it is always open, permitting sufficient examination without disturbing the limb. It is also far easier to apply, and, when applied, is self-retaining. It may be made in this way: The limb is first bandaged with wadding in roller form, enough being used to protect the bony processes and the tendo-achilles from pressure. A single layer of gauze or crinoline large enough to extend from the toes to above the knee is to be placed beneath the limb closely wrapped about it and cut so as to completely surround it with the exception of a space about an inch wide on the anterior aspect. This piece serves as a pattern by which the other layers, six or eight in all, are cut. The cloth is to be deeply slashed on each side opposite the point of the heel, to allow the foot-piece to be brought to a right angle without forming clumsy folds. The layers are now to be soaked in plaster cream, placed one upon another, applied to the limb at once, and moulded closely and carefully to it. At the sides of the ankle, where the angles from the foot-piece and the leg-piece overlap, I find it gives the neatest result if they are interlocked two at a time. A bandage rapidly applied secures a perfect fit of the splint to the limb, and can be removed when the plaster has become firmly set. If no bandage be left on the leg, the splint will accommodate itself to any reasonable amount of swelling. Some cases of Pott's fracture with marked eversion of the foot require more powerful pressure to maintain reduction than this appliance can give. For the cases, however, to the treatment of which it is adapted, it will be found a comfortable and efficient, as well as a light, safe and æsthetic dressing. While I would hesitate to advise the adoption of the spiral bandage as a routine dressing for recent fractures, I feel free to say that its advantages can be secured and its risks avoided by the use of the splint just described. Swelling may not, and in the vast majority of cases will not, occur if this be early applied. Such swelling is no more a necessary accompaniment of the repair of a fracture than of the healing of a strictly aseptic wound.

